# Long-term effects of a non-intensive weight program on body mass index and metabolic abnormalities of obese children and adolescents

**DOI:** 10.1186/1687-9856-2012-16

**Published:** 2012-06-08

**Authors:** Rita Ann Kubicky, Christopher Dunne, Debika Nandi-Munshi, Francesco De Luca

**Affiliations:** 1 Section of Endocrinology and Diabetes, St. Christopher’s Hospital for Children, Department of Pediatrics, Drexel University College of Medicine, Philadelphia, PA, USA; 2 St. Christopher’s Hospital for Children, Section of Endocrinology and Diabetes, 3601 A Street, Suite 3303, Philadelphia, PA, 19134, USA

**Keywords:** Obesity, Weight loss, Dyslipidemia, Impaired glucose tolerance, Insulin resistance

## Abstract

**Background:**

Previous studies have demonstrated positive effects of short-term, intensive weight-loss programs in obese children.

**Objectives:**

We evaluated the long-term effects of a non-intensive weight management program on the BMI, glycemic measures and lipid profiles of obese youth.

**Methods:**

Retrospective chart review of 61 obese children followed at our Weight Management Center. During visits, dietary changes and regular physical activity were recommended. Anthropometric and laboratory parameters were evaluated.

**Results:**

At the initial visit, the mean age was 11.1 ± 2.6 years. The follow-up period was 47.3 ± 11.1 months; the number of outpatient visits per year (OV/yr) was 2.9 ± 0.9. At the end of the follow-up, the whole group exhibited decreased BMI z-score and LDL-cholesterol when compared to the initial visit. In the subset of subjects in whom OGTT was performed, 2-hour glucose and peak insulin were decreased. Compared to children with ≤ 2 OV/year, those with > 2 OV/year (3.19 ± 0.7) exhibited a significant decrease in their BMI z-score, LDL-cholesterol, 2-hour glucose, and peak insulin.

**Conclusions:**

Our study suggests that a periodical (~ 3 OV/yr) evaluation in a non-intensive, long-term weight management program may significantly improve the degree of obesity and cardiovascular risk factors in childhood.

## Background

It is well known that the prevalence of obesity in children has reached epidemic proportions: during the past decade, the prevalence of children with a Body Mass Index (BMI) > 95^th^ percentile has tripled in all pediatric age-range groups [1]. Pediatric obesity is associated with significant medical complications during childhood [2], and it is a significant risk factor for morbidity and mortality in adulthood [2,3]. Most of the comorbidities of childhood obesity share insulin resistance as a common underlying mechanism. Such comorbidities (dyslipidemia, nonalcoholic fatty liver disease, type 2 diabetes mellitus (DM), hypertension, obstructive sleep apnea, polycystic ovary syndrome) tend to cluster in what is known as the metabolic syndrome [1,4].

Pediatric metabolic syndrome is a predictor of the metabolic syndrome and type 2 DM in adulthood [5]; in addition, obesity-associated atherosclerosis begins in childhood [6] and its rate of progression is greatly increased by lipid abnormalities. As a result, detecting and correcting obesity and its associated metabolic abnormalities in childhood may help prevent cardiovascular morbidity and mortality in adulthood.

A number of studies have demonstrated the positive effects of intensive weight-loss programs in children [7-11]. Yet, intensive programs are based on frequent interactions between children, their families, and a multi-disciplinary team of providers; thus, they are necessarily expensive and short-term. In addition, most of the beneficial effects of an intensive short-term intervention often do not persist once the program is completed [12-14].

Since little is known of the long-term impact of a non-intensive, conventional weight management program in obese children [15,16], we evaluated the effects of our Weight Management Program over a 4-year period at the Section of Endocrinology and Diabetes at St. Christopher’s Hospital for Children in a subset of obese patients who maintained ongoing periodic follow-up visits. The goals of our study were: 1) to analyze the changes of BMI z-score, glycemic measures and lipid profiles at the end of the 4-year follow-up period, and 2) to correlate these changes with the frequency of the follow-up visits.

## Methods

We conducted a retrospective analysis of the medical records of obese (BMI > 95^th^ percentile for age and sex) children and adolescents evaluated between 2001 and 2008 at the Weight Management Center in the Section of Endocrinology and Diabetes at St. Christopher’s Hospital for Children. All children were identified by tracking patients for whom the ICD-9 Code 783.1 was used (“abnormal weight gain”). Most of these patients were referred to us by their pediatricians for an evaluation of overweight/obesity and/or high serum insulin/abnormal lipid levels.

Inclusion criteria were the following: [1] children with a BMI > 95^th^ percentile, [2] 1–18 years of age [3] ≥ 2 years of follow-up, and [4] fasting lipid panel, glucose and insulin obtained at the beginning and at the end of the follow-up period. Exclusion criteria included: 1) diagnosis of DM (type 1 or type 2) and 2) use of medications known to affect insulin sensitivity or glucose/lipid metabolism (metformin, insulin, growth hormone).

The Institutional Regulatory Board of Drexel University College of Medicine approved the retrospective analysis of the medical records.

At the initial visit, a physician screened the obese subjects for metabolic comorbidities, while a registered dietitian assessed their dietary habits and amount of physical activity. Generalized handouts geared towards healthy eating were provided to each subject; the dietitian reviewed each topic of the handout with the subjects and their parent/guardian. The topics included: making healthy food choices (increasing whole grains, lean meats and lower fat foods into the diet), eating three balanced meals per day using the plate method, portion control (measuring portions and learning about food labels), eliminating beverages containing more than 5 calories except for low fat milk, and making sensible snack choices. Thirty minutes of daily physical activity was recommended to all subjects with a focus on an activity that the child would enjoy.

At the subsequent visits (scheduled every 2 to 4 months), obese children and adolescents met with a pediatric nurse practitioner and the dietitian for approximately 20 minutes with each of the two providers; in order to measure implementation of the previous recommendations, during each of the follow-up visits, the child’s dietary intake and physical activities were reassessed by patient history and diet and exercise recall. Patients who reported adhering to the dietary or physical activity changes were praised and encouraged to continue. If there was poor adherence to previous recommendations, or if there was need for further improvement, efforts were made by the dietitian and nurse practioner to detect barriers to change and to help determine alternative ways of engaging patients to adhere.

At each visit, body weight was measured with a balance scale, and height was measured with a wall-mounted stadiometer by a trained medical assistant. BMI was calculated as weight in kilograms divided by the height in meters squared, and expressed as a z-score by using the Centers for Disease Control and Prevention 2000 program [17]. Body weight, height and BMI were compared to the measurements/calculations of the previous visit by the nurse practitioner and dietitian in order to monitor each patient’s weight loss/gain and change in the BMI as standard of care, and as an objective way to measure the likelihood that the patient was adhering to the dietary and physical activity-related recommendations.

A fasting lipid panel (total cholesterol, low-density lipoprotein [LDL] cholesterol, high-density lipoprotein [HDL] cholesterol, and triglyceride [TG] levels) was requested yearly. A 2-hour Oral Glucose Tolerance test (OGTT) was recommended to all children: impaired glucose tolerance (IGT) and DM were defined as a 2-hour glucose level of 140–199 mg/dL and ≥ 200 mg/dL, respectively [18]. Insulin resistance was estimated by using the Homeostasis Model Assessment of Insulin Resistance (HOMA-IR), calculated as fasting plasma glucose (mg/dL) x fasting insulin (μU/mL) ÷ 405 [19]. Dyslipidemia was defined as high TG (≥ 90^th^ percentile for age and sex) and/or low HDL-cholesterol (≤ 10^th^ percentile for age and sex) concentrations [4].

Data were analyzed with SPSS software version 17.0 for Windows (SPSS, Chicago, IL). All data were expressed as the mean plus or minus SD or range. A p-value < 0.05 was considered to be statistically significant. Differences in the mean values between groups were evaluated using a Student’s *t*-test or analysis of variance.

## Results

A total of 61 children and adolescents met our inclusion criteria. 39 children were females and 24 were prepubertal; the mean age was 11.1 ± 2.6 years (mean ± SD). With respect to their ethnicity, 25 were African American, 26 were Hispanic, 7 were Caucasian, 2 Asian and 1 identified himself as other. The duration of the follow-up period was 47.3 ± 11.1 months while the number of outpatient visits per year (OV/yr) was 2.9 ± 0.9.

At the end of the follow-up, children exhibited a significant decrease in their BMI z-score (p = 0.03) and LDL-cholesterol (p = 0.022) (Table 1). In the subset of children in whom OGTT was performed both at the beginning and at the end of the follow-up period (n = 42), there was a significant decrease in both the 2-hour glucose (p = 0.004) and peak insulin (p = 0.043) (Table 1).

**Table 1:** Anthropometric and metabolic characteristics of the population sample

	**Initial Visit**	**Last Visit**	**p-value**
BMI z-score	2.49 ± 0.4	2.33 ± 0.4	**0.03**
Fasting glucose (mmol/L)^a^	4.8 ± 0.5	4.6 ± 0.5	0.08
Fasting insulin (pmol/L)^b^	145.1 ± 90.3	135.4 ± 100.7	0.56
HOMA-IR	4.5 ± 3	4.1 ± 2.9	0.38
HDL-cholesterol (mmol/L)^c^	1.2 ± 0.3	1.2 ± 0.3	0.61
LDL-cholesterol (mmol/L)^c^	2.9 ± 0.9	2.5 ± 0.6	**0.022**
Triglycerides (mmol/L)^d^	2.8 ± 1.8	2.3 ± 0.9	0.057
2-hour glucose (mmol/L)^a^	2.9 ± 0.6	2.5 ± 0.7	**0.004**
Peak insulin (pmol/L)^b^	1445.9 ± 869.5	1070.9 ± 748.7	**0.043**

Results are expressed as mean ± SD. p-values < 0.05 are in bold.

^a^To convert to mg/dL divide by 0.0555.

^b^To convert to μIU/mL divide by 6.945.

^c^To convert to mg/dL divide by 0.0259.

^d^To convert to mg/dL divide by 0.0113.

All children with an initially normal OGTT (n = 37) maintained a normal OGTT by the end of the follow-up period, with the exception of 1 child who developed IGT (2-hr glucose, 141 mg/dL); his HOMA-IR increased and BMI z-score decreased. 5 children were found with IGT on their initial OGTT; their BMI z-score and HOMA-IR were similar to those of children with normal OGTT. In 4 children with IGT, the OGTT normalized by the end of the follow-up, while the 5^th^ child developed DM. Of the 4 children with normalized OGTT, 1 child experienced increased BMI z-score and HOMA-IR, 1 child decreased BMI z-score and increased HOMA-IR, and 2 children increased BMI z-score and decreased HOMA-IR. The child who became diabetic had decreased BMI z-score and HOMA-IR by the end of the follow-up period.

When children were grouped according to changes in BMI z-score [increased vs. same/decreased BMI z-score (0.2 ± 0.15 vs. -0.32 ± 0.3, p < 0.001)], those with the same or decreased BMI z-score exhibited decreased fasting insulin (136.7 ± 98.6 vs. 153.5 ± 91 pmol/L, last vs. initial visit, p < 0.001) and LDL-cholesterol (2.4 ± 0.5 vs. 2.7 ± 0.9 mmol/L, last vs. initial visit, p = 0.02).

Among the 60 subjects for whom a fasting lipid panel was obtained at the initial visit, 25 children had dyslipidemia: 5 with low HDL and high TG, 11 with isolated low HDL and 9 with isolated high TG; when compared to those with normal lipid levels, children with abnormal levels had similar BMI z-scores (Table 2). At the end of the follow-up period, lipid levels normalized in 15 children and remained abnormal in 10 children: the 15 children with normalized lipid levels exhibited a significantly decreased BMI z-score (Table 3), unlike those with persistently abnormal lipid profile.

**Table 2:** Comparison of BMI z-score and metabolic parameters according to the presence of dyslipidemia at the initial visit

	**Dyslipidemia** **(n=25)**	**Normal Lipids** **(n=35)**	**p-value**
BMI z-score	2.42 ± 0.4	2.51 ± 0.4	0.42
HDL-cholesterol (mmol/L)^a^	1.0 ± 0.2	1.3 ± 0.2	**< 0.001**
Triglycerides (mmol/L)^b^	1.6 ± 1.0	0.9 ± 0.3	**< 0.001**
HOMA-IR	4 ± 1.8	4.6 ± 3.1	0.45

Results are expressed as mean ± SD. p-values < 0.05 are in bold.

^a^To convert to mg/dL divide by 0.0259.

^b^To convert to mg/dL divide by 0.0113.

**Table 3:** Changes of BMI z-score and metabolic parameters at the last visit according to changes of lipid levels

	**Abnormal HDL & TG** **↓** **Abnormal HDL & TG** ** (n=10)**		**Abnormal HDL & TG** **↓** **Normal HDL & TG** ** (n=15)**		**Normal HDL & TG** **↓** **Abnormal HDL & TG** **(n=5)**		**Normal HDL & TG** **↓** **Normal HDL & TG ** **(n=30)**	
	**Initial Visit**	**Last Visit**	**Initial Visit**	**Last Visit**	**Initial Visit**	**Last Visit**	**Initial Visit**	**Last Visit**
BMI z-score	2.36 ± 0.4	2.32 ± 0.5	2.49 ± 0.4	2.19 ± 0.3*	2.47 ± 0.5	2.47 ± 0.5	2.52 ± 0.4	2.37 ± 0.4
HDL-cholesterol (mmol/L)^a^	0.9 ± 0.1	0.9 ± 0.1	1.1 ± 0.3	1.1 ± 0.2	1.2 ± 0.1	1 ± 0.1*	1.3 ± 0.2	1.3 ± 0.3
Triglycerides(mmol/L)^b^	1.9 ± 1.4	1.3 ± 32	1.6 ± 0.7	1 ± 0.3*	1 ± 0.3	1.3 ± 0.4	0.9 ± 0.3	0.9 ± 0.3
HOMA-IR	4.2 ± 1.9	5.3 ± 3.3	4.7 ± 3.4	3.7 ± 2	4 ± 2.4	3.3 ± 2.4	4.7 ± 3.2	3.9 ± 3.2

Results are expressed as mean ± SD. *p-value < 0.05, last vs. initial visit.

^a^To convert to mg/dL divide by 0.0259.

^b^To convert to mg/dL divide by 0.0113.

5 children with normal HDL and TG at baseline developed dyslipidemia at the end of the follow-up period: 1 child developed both elevated TG and low HDL, 3 children had low HDL and 1 developed high TG. These 5 children did not experience any significant change of the mean BMI-z-score or HOMA-IR at the end of the follow-up period (Table 3).

When children were divided in two groups according to their mean number of OV per year (≤ 2 vs. >2; 1.53 ± 0.5 vs. 3.19 ± 0.7, p < 0.001), there were no differences at the initial visit for any of the metabolic variables analyzed or for BMI z-score (Table 4). At the end of the follow-up, children with > 2 OV/yr exhibited a significant decrease in their LDL-cholesterol (Table 4) and BMI z-score (Table 4), while children with ≤ 2 OV/yr did not. In the group of children who underwent OGTT, those with > 2 OV/yr experienced a significant decrease of both the 2-hour glucose (n = 33), (Table 4, p = 0.0001) and peak insulin (n = 32), (Table 4, p = 0.0036), while children with ≤ 2 OV/yr did not (n = 8 and 6, respectively).

**Table 4:** Changes of BMI z-score and metabolic parameters according to the frequency of clinic visits

	**≤ 2 Outpatient visits ** **(n=13)**		**p-value**	**>2 Outpatient visits** **(n=48)**		**p-value**
	**Initial Visit**	**Last Visit**		**Initial Visit**	**Last Visit**	
Age (years)	10.9 ± 1.64			11.2 ± 2.9		0.71
Sex (male/female)	5/8			17/31		0.84
BMI z-score	2.44 ± 0.4	2.37 ± 0.4	0.66	2.5 ± 0.4	2.32 ± 0.4	**0.03**
Fasting glucose (mmol/L)^a^	4.8 ± 0.32	4.5 ± 0.4	0.09	4.8 ± 0.6	4.7 ± 0.5	0.21
Fasting insulin (pmol/L)^b^	151.4 ± 59.0	150.01 ± 95.8	0.97	143.8 ± 97.2	131.3 ± 102.1	0.54
HOMA-IR	4.7 ± 1.8	4.4 ± 2.9	0.8	4.5 ± 3.2	4 ± 2.9	0.4
HDL-cholesterol (mmol/L)^c^	1.2 ± 0.2	1.1 ± 0.3	0.25	1.2 ± 0.3	1.2 ± 0.3	0.98
LDL-cholesterol (mmol/L)^c^	106.2 ± 84.4	97.9 ± 12.3	0.16	111.3 ± 36.7	96.4 ± 23.5	**0.022**
Triglycerides (mmol/L)^d^	1.6 ± 1	1.2 ± 0.4	0.20	1.1 ± 0.7	1 ± 0.4	0.13
2-hour glucose (mmol/L)^a^	(n=8) 5.8 ± 1.2	5.3 ± 1.3	0.44	(n=34) 6.3 ± 1.2	5.2 ± 1.0	**0.0001**
Peak insulin (pmol/L)^a^	(n=6) 1486.9 ± 1039.0	1474.4 ± 1206.3	0.98	(n=32) 1472.3 ± 817.4	952.9 ± 563.2	**0.0036**

Results are expressed as mean ± SD. p-values < 0.05 are in bold.

^a^To convert to mg/dL divide by 0.0555.

^b^To convert to μIU/mL divide by 6.945.

^c^To convert to mg/dL divide by 0.0259.

^d^To convert to mg/dL divide by 0.0113.

## Discussion

In our multi-ethnic population sample, a non-intensive (~ 3 visits per year) weight management program that reinforced healthy dietary modifications and regular daily activity over a 4-year period resulted in a statistically significant reduction of BMI z-score and LDL-cholesterol, and improvement of glucose tolerance.

Extensive evidence previously published supports the effectiveness of intensive weight-loss programs in children. In a study conducted by Wilfley et al., 204 overweight children were enrolled to determine the short-term and long-term efficacy of weight-loss and weight maintenance programs [7]. After 5 months of intensive weekly meetings focused on weight-loss treatment with a multi-disciplinary team, almost 90% of children exhibited a decreased BMI z-score. At the end of the weight-loss intervention, the 2 active maintenance groups experienced a mean change in BMI z-score of – 0.22 from baseline to 2-year follow-up versus the control group. Such BMI z-score reduction is similar to the one shown in our study by the end of the 4-year follow-up (−0.16); however, Wilfley et al. did not evaluate the impact of weight loss on metabolic parameters. Savoye et al. studied a population of 209 obese children to evaluate the effects of a 12-month weight management program on adiposity and metabolic parameters [8]. The program included exercise, nutrition, and behavior modification: intervention occurred biweekly the first 6 months and bimonthly thereafter. At the end of study, the weight-management group experienced a significant decrease of BMI and HOMA-IR compared to the control group; conversely, no difference was found relative to changes in fasting glucose, HDL-cholesterol, LDL-cholesterol, or blood pressure. Reinehr et al. studied changes in weight status and cardiovascular disease (CVD) risk factors in 203 obese children who attended a 1-year outpatient intervention program; enrolled subjects were then evaluated 1 year after the end of the intervention [20]. The program included weekly meetings with an exercise physiologist as well as once to twice monthly with a dietitian and a psychologist. Children who experienced a reduction of BMI SDS (72% of the group) at the end of the 12-month intervention maintained this reduction 1 year later. In addition, children with reduced BMI SDS (but not those without) showed improved HDL-cholesterol, LDL-cholesterol, blood pressure, and HOMA-IR.

Although the positive effects of all these studies were sustained for a relatively long period of time, the high costs associated with the frequent utilization of a team of dietitians, social workers, and exercise physiologists render this format not widely applicable. In contrast, our findings suggest that weight management programs based on less frequent encounters with a smaller team (pediatric nurse practitioner and a registered dietitian) may result in a similarly effective and lasting reduction of obesity and obesity-associated metabolic abnormalities.

The importance of preventing or reducing the severity of overweight in childhood is supported by a number of studies demonstrating the link between pediatric obesity and morbidity and mortality in adulthood. Three previous studies have identified an association between overweight in children and adolescents with increased rates of death due to coronary heart disease [21,22] and with death from all causes [22,23]. In a large cohort of American-Indian subjects followed since childhood [3], the rate of premature death (before 55 years of age) from endogenous causes among children in the highest quartile of BMI was more than double than that in children in the lowest quartile. Of note, the association between BMI and premature death was attenuated but remained significant after adjustment for glucose level, cholesterol level, and blood pressure: thus, some of the effects of overweight on the risk of premature death may not depend on abnormal glucose and lipid metabolism, or on hypertension.

In our cohort of 61 children, 5 were found with IGT at baseline; in 4 of these children, the OGTT normalized by the end of the 4-year follow-up period, while one child became diabetic. In a similar study, Weiss et al. identified 33 children with IGT in a population sample of 117 obese children [24]. By the end of a 2-year period, 15 of those with IGT reverted to normal glucose tolerance while 8 developed Type 2 DM. When compared to those who reverted to normal glucose tolerance, subjects who developed DM were significantly more obese at baseline and increased their BMI during the follow-up period; in our study, the relationship between OGTT results and initial BMI z-score and/or change in BMI z-score overtime is less clear. While the association between IGT and risk to develop DM has not been well defined in children, it has been clearly demonstrated in adults [25,26]; in addition, IGT in adults appears to be linked to an increased risk for cardiovascular disease and mortality [27,28].

In the present study, 25 of the 60 subjects had dyslipidemia at the initial visit: by the end of the 4-year follow-up period, in 15 of these 25 subjects the abnormal lipid levels normalized: unlike those with persistently abnormal lipid panel, the 15 children with normalized lipid levels exhibited a significantly decreased BMI z-score during the 4-year follow-up period. Previous cross-sectional studies have shown a high prevalence of low HDL-cholesterol and elevated TG in obese children [29,30]. A longitudinal study conducted in the United Kingdom in more than 5,000 children showed that 1 SD greater BMI at age 9–12 years was associated with high TG and low HDL-cholesterol at age 15–16 years [31]; in the same study, changing from overweight/obese at age 9–12 to normal weight at age 15–16 was associated with better cardiovascular risk profiles than remaining overweight/obese from childhood through adolescence. Data from 4 prospective cohorts have demonstrated that cardiovascular risk factors in childhood (including high TG) significantly predict subclinical atherosclerosis as early as 9 years of age [32], thus justifying sustained efforts to correct obesity and lipid abnormalities in children.

There are some limitations of our study, such as the lack of a control group and the relatively small sample size. However, the fact that our results are consistent with those of studies including a larger number of subjects and a control group supports the validity of our findings. In addition, there may have been a selection bias regarding the subjects included in the retrospective analysis, since only a small number of children initially evaluated at our Weight Management Center were eventually followed for 2 or more years. We can speculate that the effects of the program were less significant for those subjects followed for less than 2 years. Those subjects having a longer duration of follow-up may have experienced weight loss early in the program, greater adherence to the lifestyle changes, or more family involvement. Our results demonstrate that a non-intensive weight management program offers potential medical benefits to children and adolescents who are sufficiently motivated to continue their follow-up visits.

The long duration of our retrospective study, and the non-intensive approach of our intervention, has rendered unfeasible the concomitant evaluation of a control, completely untreated, group of obese children. To circumvent such limitation, we have used two historical control groups followed longitudinally by Reinehr et al. [33] and by D’Hondt et al. [34]. In the former study, 100 overweight children [BMI-SDS 1.92 (1.27-2.75)] with a mean age of 9 years (6–15 years) were periodically evaluated during a 2-year period without any intervention. This control group did not experience any significant change in their BMI-SDS. In the latter study by D’Hondt et al., at baseline 50 overweight children (8 of which were obese) had a mean age of 11.6 ± 0.8 years and a baseline BMI z-score range of 1.55 ± 0.39 (1.00; 2.64). 2 years later, even these children’s BMI z-scores remained unchanged. These finding suggests that the significant reduction of BMI-SDS observed in our study likely depends on the lifestyle modifications reinforced by our team, rather than simply reflecting a physiological change in adiposity.

In conclusion, our study suggests that a non-intensive, long-term weight management program may significantly improve the degree of obesity and some cardiovascular risk factors in childhood. In addition, this non-intensive treatment (a small team approach) is more likely to be reimbursed by 3^rd^ party payors making it more financially sustainable. Prospective studies with a larger population sample and comparison to a control group are warranted to confirm these findings.

## Abbreviations

BMI: Body Mass Index; OV/yr: Outpatient visits per year; DM: Diabetes Mellitus; HDL: High-density lipoprotein; LDL: Low-density lipoprotein; TG: Triglycerides; OGTT: Oral Glucose Tolerance Test; IGT: Impaired glucose tolerance; HOMA-IR: Homeostasis Model Assessment of Insulin Resistance; CVD: Cardiovascular disease.

## Competing interests

The authors disclose no potential, perceived, or real conflict of interest.

## Authors’ contributions

DN and CD initiated data collection; CD continued data collection and started literature search. RAK continued data collection and completed literature search with FDL. CD, RAK and FDL analyzed data. RAK and FDL as well as CD wrote the paper. All authors had final approval of the submitted version.
